# Expression Levels of Thymidylate Synthase, Thymidylate Phosphorylase and Dihydropyrimidine Dehydrogenase in Head and Neck Squamous Cell Carcinoma: Preliminary Study

**Published:** 2008-01-22

**Authors:** K. Aubry, J.L. Labourey, J.P. Bessède, N. Tubiana-Mathieu, M. Rigaud

**Affiliations:** 1 E.N.T. Department, University Hospital Center, 2 Avenue Martin Luther-King, 87000 Limoges, France; 2 Oncology Department, University Hospital Center, 2 Avenue Martin Luther-King, 87000 Limoges, France; 3 Biochemistry Department, University Hospital Center, 2 Avenue Martin Luther-King, 87000 Limoges, France

**Keywords:** 5-FU, genes of sensitivity, chemotherapy, squamous cell carcinoma, pharyngolarynx

## Abstract

**Introduction:**

Pharyngo-laryngeal tumors classified as T3-4, N0-3, M0, are conventionally treated by mutilating surgery (total (pharyngo)-laryngectomy). Neo-adjuvant chemotherapy with 5-FU/platinum salt can be proposed in an attempt to preserve the larynx. The level of the response to chemotherapy ranges from 36 to 54% of cases. Thus, a large number of patients receive chemotherapy that is ineffective and not free from adverse effects. Three main enzymes are involved in the metabolism of 5-FU: thymidylate synthase (TS), thymidylate phosphorylase (TP) and dihydropyrimidine dehydrogenase (DPD). Several studies suggest that a high level of expression of these three genes correlates with a poor clinical response to 5-FU. The main purpose of our study was to look for a correlation between the levels of expression of the genes for sensitivity to 5-FU (TS, TP, DPD) within the tumor and the clinical response observed after three courses of chemotherapy combining 5-FU/platinum salt in patients presenting with advanced cancer of the pharyngo-larynx.

**Methods:**

This was a prospective genetic study that had required approval from the Ethics Committee. The main assessment criterion was based on the assessment of the clinical response by an ENT panendoscopy and a cervical CT scan, after three courses of chemotherapy. The expression of the genes was determined by quantitative RT-PCR, using total RNA extracted from tumor biopsies taken during the initial panendoscopy.

**Results:**

The means calculated, in our study, for the three genes of interest (TS, TP, DPD) were lower in the responder group than those in the non-responder group.

**Discussion:**

Our preliminary findings reveal trends that confirm the hypothesis that the lower the level of expression of the sensitivity genes, the better the clinical response to chemotherapy. They now form part of a larger study that is currently in progress.

## Introduction

Over the past 20 years, advances in the treatment of pharyngo-laryngeal epidermoid carcinomas have made it possible to reduce the functional impact on swallowing, breathing and speaking. Cancers classified as T3 or T4, N0-3, and M0 are conventionally treated by total (pharyngo)-laryngectomy (T(P)L). For the past few years, it has been possible to propose combined chemotherapy with 5-fluoro-uracyl (5-FU) plus a platinum salt (Cisplatin or Paraplatin), thus preserving the larynx. This chemotherapy produces a regression of the lesions in 36 to 54% of cases (1, 2). 5-FU is an anti-metabolite with a chemical structure analogous to that of pyrimidine bases. Three essential enzymes are implicated in its metabolism: thymidylate synthase (TS), thymidylate phosphorylase (TP) and dihydropyrimidine dehydrogenase (DPD). TS is an essential enzyme in the synthesis of DNA, and when it is blocked by a metabolite of 5-FU (5-FdUMP) this results in the inhibition of the synthesis of DNA. TP, an enzyme involved in the anabolism of 5-FU, is also involved in tumor neoangiogenesis (3). DPD is an enzyme involved in the catabolism of 5-FU. Patients with low DPD activity are unable to activate 5-FU effectively, which results in the accumulation of toxic active metabolites. The mechanisms of resistance to 5-FU may either be present prior to the treatment or be acquired during treatment. Several possible mechanisms have been proposed, but their real clinical involvement remains to be demonstrated. Studies of colo-rectal and gastric adenocarcinomas have demonstrated that the expression of the genes depends on the tumors involved, and there is a correlation between the expression of the TS, TP, and DPD genes and their translation into proteins for a given tumor (4, 5). According to several studies, the levels of expression of mRNA of these three enzymes in colon tumors are indicators that predict the sensitivity to 5-FU (6–8), and their over-expression within the tumor is one of the mechanisms of resistance to 5-FU (9). There have been few studies of ENT cancers, and their findings are contradictory. A study of cancers at several sites in the head and neck highlights the importance of determining the expression of TS in order to identify the patients who could benefit from 5-FU treatment (10), something not confirmed by two more recent studies (11, 12). In the case of DPD, the study by Kawasaki et al. (11) demonstrates a statistically significant relationship between the expression of DPD in the tumor and the clinical response to 5-FU, a relationship not found in the study by Etienne et al. (12). No study of TP in ENT cancers has been published.

The reverse transcription and quantitative real-time polymerase chain reaction (RT-PCR) techniques make it possible to determine the expression of various genes using small tumor fragments.

The aim of our study was to look for a relationship between the levels of expression within the tumor of the genes for 5-FU sensitivity (TS, TP, DPD) and the clinical response observed after three courses of chemotherapy using a combination of 5-FU/platinum salt, in T3-4, N0-3, M0 pharyngolaryngeal cancers. The clinical aim was to be able to identify patients who would not respond to the chemotherapy, in order to propose radical surgery from the outset.

## Materials and Methods

### Materials

#### Type of study

This was a prospective, single-center study with no direct individual benefit, carried out over a period of 6 months. The protocol of the study has been submitted to the Ethics Committee.

#### Patient inclusion criteria

The patients presented with an operable epidermoid carcinoma of the larynx or hypopharynx, classified as T3 or T4, N0-3, M0 (TNM classification) for which it had been decided there was a therapeutic indication for neoadjuvant chemotherapy, consisting of three courses of a combination of 5-FU/platinum salt. Each course lasted 5 days. The interval between two successive courses was 21 days (D1= DJ21). The doses administered during each course were 100 mg/m^2^ of Cisplatin on D1, and a continuous infusion of 1000 mg/m^2^/d of 5-FU from D1 to D5. The patients’ general condition was compatible with the introduction of chemotherapy (WHO *Performance Status (PS) Scale* ≤ 2 or *Karnofsky Index >* 60%). The patients were over 18 years of age. They had been informed about the study, and had signed an informed consent form.

#### Biological material

Tumor biopsies were taken during the initial panendoscopy. The pathologist placed them in cryo-tubes and then froze then at −80 °C in the tumor archive.

### Methods

#### Initial TNM classification

This was carried out after a panendoscopy and a cervical tomodensitometry.

#### Experimental protocol

The determination of the expression of the genes for sensitivity to 5-FU (TS, TP, DPD) within the tumor was carried out in three successive steps: extraction of the total RNA, reverse transcription and real-time polymerase chain reaction (PCR).

##### RNA extraction

This was carried out using the principle of the RNeasy kit, which is based on selective binding of RNA to silica gel in columns. Frozen tumor biopsy samples (30 mg) were ground in liquid nitrogen with steel ball bearings, using a tissue mill (Mixer Mill MM 300, Qiagen). The ground tissue was then subjected to lysis in a guanidine isothiocyanate buffer, to denature the proteins and inactivate the RNases. The lysate obtained was mixed with ethanol, which produced selective binding of the RNA to a membrane containing silica gel. In order to ensure the purity of the RNA, an additional digestion step using DNase I was included in the protocol. The bound RNA was then eluted using «RNase-free» water. The quality of the RNA was systematically checked by migration on agar gel (electrophoresis), and the quantity obtained was determined by spectrophotometric assay. Any RNA that appeared to be damaged during the electrophoresis was routinely excluded from the analysis.

##### Reverse transcription

This is used to transcribe RNA into complementary DNA (cDNA) using inverse transcriptase from an oligo-d(T)_15_, which has the property of forming a hybrid with the poly-A tail of eucaryotic mRNA. The enzyme used in our study was AMV reverse transcriptase (Avian-Myeloblastosis-Virus). Each reverse transcription was carried out using 0.5 μg of mRNA. The mRNA was exposed to 20 U of AMV reverse transcriptase, 10 mM of dNTP, 1.6 μg of oligo d(T)_15_ primers and of 40 U of RNase inhibitor, according to the following sequences: 10 minutes at 25 °C, 1 hour at 55 °C and 5 minutes at 94 °C. For each sample, an RT-negative (RT-) and RNA-negative (RNA-) control was carried out to confirm the absence of DNA contamination in the RNA extract, and the absence of contamination of the mix used for the RT reaction respectively.

##### Real-time polymerase chain reaction (PCR)

This method is used to monitor the amplification of the candidate gene as the reaction progresses. It uses a fluorochrome, SYBR GreenI, which is incorporated in a small channel in double-stranded DNA. Specific software for the Light Cycler was used to measure the total fluorescence, which is proportional to the number of molecules of the amplified gene. In addition, the presence of SYBR GreenI in the reaction medium makes it possible to plot the fusion curves for the double-stranded DNA, and thus to confirm the specificity of the amplification characterized by the Tm (fusion or denaturing temperature of the DNA fragment). A pair of specific primers was used for each gene of interest (Table 1). The dilution range was prepared from external standards consisting of RNA extracts from a colo-rectal cancer cell line (WiDr). The RNA obtained in this way was then retro-transcribed into cDNA, before being amplified using the Light Cycler under the same conditions as those used for the cDNA from the samples in the tumor archive. The standards were used to produce a range of dilutions of cDNA. Each transcription product was amplified without diluting (pure), and after diluting tenfold (d10), one-hundred fold (d100) and one thousand fold (d1000). The higher the concentration of the cDNA of the gene of interest in the sample, the earlier the amplification from the specific primers started. This made it possible to plot a regression line to determine the initial concentration of the mRNA of the gene of interest. In order to confirm the values obtained, each sample was measured in duplicate and twice for each gene. The mean of the four values obtained was calculated. Only results within the standard range were accepted. Contamination was detected as the presence of DNA in the blank and in the RT- and RNA-controls. A reference gene (glucose-6-phosphate dehydrogenase (G6PDH)) was required for the purpose of quantification. Its role was to ensure that there were no measurement errors due to any pipetting errors, discrepancies in the assay of the matrix or the RT reactions.

The ratio of the concentration of mRNA of the gene of interest (TS, TP, DPD) over the concentration of the mRNA of the reference gene was used to determine the level of expression of the mRNA of the gene of interest.

#### Main assessment criterion

The main assessment criterion was based on the clinical response after three courses of chemotherapy. This was assessed by a panendoscopy and a cervical tomodensitometry, during the 2 weeks following the third course of chemotherapy. The two panendoscopies (initial and after the 3rd course) were carried out by the same operator. The clinical response was determined objectively, according to the WHO criteria (Table 2), and the patients were then divided into two groups: the responder group (R), consisting of the patients displaying a complete response (RC) or a partial response (RP), and the non-responder group (NR), consisting of the patients with progressive (PD) or stable (SD) disease.

#### Statistical analysis

The statistical analysis consisted of calculating the means and standard deviations of the degree of expression of the mRNA of the genes of interest, in the responder (R) and non-responder (NR) groups.

## Results

**Figure 1:**
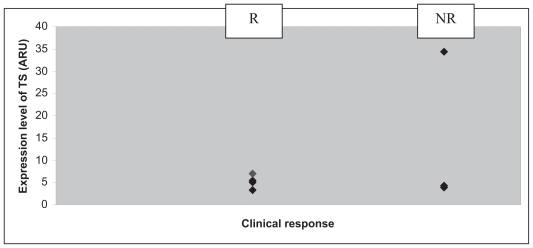
Level of TS mRNA (♦) in arbitrary relative units (ARU) depending on clinical response after C3: patient responder (R) or non-responder (NR).

**Figure 2:**
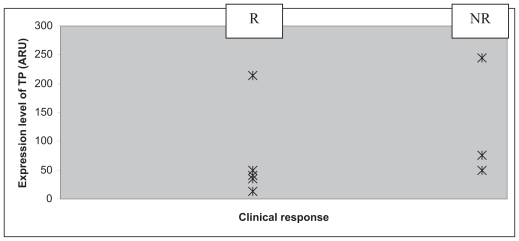
Level of TP mRNA ( 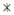) (ARU) depending on clinical response after C3: patient responder (R) or non-responder (NR).

**Figure 3:**
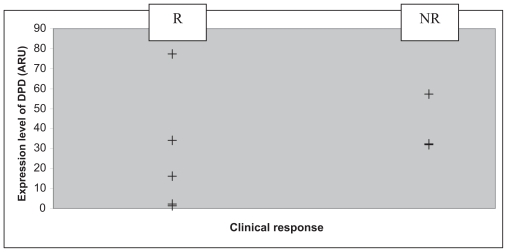
Level of DPD mRNA (+) (ARU) depending on clinical response after C3: patient responder (R) or non-responder (NR).

All the patients included were men (Table 3). Their group mean age was 58.5 years. Five patients presented with a lesion of the larynx, and five with one of the hypopharynx. With regard to their histology, four patients presented with a clearly differentiated epidermoid carcinoma and six patients with a carcinoma that was only moderately or slightly differentiated. The patients were all given three courses of chemotherapy with 5-FU plus a platinum salt, apart from three patients who displayed disease progression during chemotherapy (numbers 2, 7 and 8). One of these three patients showed disease progression at the end of the third course of chemotherapy that led to his being classified as inoperable, and so a fourth course of chemotherapy was administered followed by radio-therapy. The second patient displayed disease progression, making it necessary to carry out an emergency tracheotomy, followed by surgery (total pharyngo-laryngectomy), two weeks after the end of the first course of chemotherapy. The third patient displayed secondary lesions on his lungs after C2, which led to the continuation of the courses of chemotherapy combined with radio-therapy. The clinical outcome consisted of 5 partial responses, one complete response, one case in which the disease remained stable and three cases of disease progression (Table 4). We saw only one case in which the organ was preserved after a complete response. All the responder patients were classified as T3. The levels of TS, TP and DPD mRNA within the tumor were measured in each patient. The lowest level of expression of TS was 3.34 ARU (arbitrary relative units), and the highest 34.45 ARU (Fig. 1). The mean values obtained in the responder (R) and non-responder (NR) groups were 5.61 ARU [3.34; 9.55] and 12.52 ARU [4.33; 34.45] respectively. The standard deviations for the R and NR groups were 2.37 and 14.70 respectively. The lowest level of expression of TP was 12.93 ARU, and the highest 245.15 ARU (Fig. 2). The mean values obtained in the R and NR groups were 66.31 ARU [12.93; 214.79] and 96.27 ARU [15.09; 245.15] respectively. The standard deviations for the R and NR groups were 73.83 and 102.27 respectively. The lowest level of expression of DPD was 1.35 ARU, and the highest 77.2 ARU (Fig. 3). The mean values obtained in the R and NR groups were 25.04 ARU [1.35; 77.2] and 30.68 ARU [1.36; 57.33] respectively. The standard deviations in the R and NR groups were 28.30 and 22.90 respectively.

## Discussion

Our study was intended to look for a correlation between the levels of expression of the genes for sensitivity to 5-FU (TS, TP, DPD) within the tumor and the clinical response to the chemotherapy, consisting of a combination of 5-FU plus a platinum salt, in patients with a cancer of pharyngolarynx classified as T3-4, N0-3, M0. These preliminary findings are part of a wider study currently in progress. Although we do not provide any significant results, there does seem to be a trend that supports the initial hypothesis that the higher the levels of expression of the mRNA of TS, TP, DPD within the tumor, the lower the clinical response to chemotherapy. The mean values obtained in our study for the 3 genes of interest (TS, TP, DPD) were lower in the R group than in the NR group. According to some studies, these 3 genes for sensitivity to 5-FU are independent factors (9, 11), which could explain why some patients were classified as responders even though they displayed a high level of expression of one of these genes.

The literature includes only 4 studies concerning the genes for sensitivity to 5-FU in ENT cancers (10–13). Three of these 4 studies were prospective studies, and one was a retrospective study. They included far greater numbers of patients: 109 (10), 62 (11), 82 (12) and 86 (13) respectively, but were carried out over several years. In our study, we chose to include only patients presenting with an epidermoid carcinoma of the pharyngolarynx, in contrast to the other studies (10, 12, 13), which had included a wide variety of cancers of the head and neck. Kawasaki et al. (11) included only epidermoid carcinomas of the buccal cavity. Furthermore, these studies included all tumor stages (T1-4), whereas we only included advanced stages of pharyngolaryngeal cancer (T3-4). Our study had a specific clinical objective, that of being able to select patients likely to respond to neo-adjuvant chemotherapy, with the aim of preserving the larynx. We did not include any patients classified as inoperable and who were receiving treatment with a combination of chemotherapy (5-FU/platinum salt)-radiotherapy. This was in order to enable us to differentiate between the effects of chemotherapy and radiotherapy on the clinical response, and so avoid bias in the analysis of the results, unlike the study of Etienne et al. (12). The chemotherapy protocol used in our study was the same as that used by Etienne et al. (12, 13) but different from that of Johnston et al. (10). Consequently, our work is difficult to compare to that of other authors. We worked on tumor biopsies taken during the initial panendoscopy, under visual control, in the same way as Etienne et al. (12). This technique has its limitations, since the sample may have been taken from a heterogeneous fragment of tumor or at the edge of the tumor, and so contain healthy cells. In order to improve the reliability of the sample, it would be useful to use the laser microdissection method, which makes it possible to isolate tumor cells within a heterogeneous tissue.

We studied the level of DPD mRNA in the tumor. Some studies stress the value of determining this level not only in the tumor, but also in the healthy tissue around the tumor (11, 12). According to these publications, it is only the ratio (of the expression of DPD in the tumor to that in the non-tumor tissue) that correlates to the response to chemotherapy. In our study, no healthy tissue could be taken for ethical reasons.

We chose to measure the levels of expression of the genes for sensitivity to 5-FU, using the quantitative RT-PCR technique. This is a method that is suitable for use with small samples, such as our tumor biopsies. It is also quantitative and readily reproducible, unlike immunohistochemistry and enzymology methods. Immunohistochemistry is a qualitative technique that involves at least two different observers (10, 11). Enzymology requires larger tumor samples, and it is also more fastidious and less reproducible than RT-PCR (12, 13). However, these two techniques directly involve the protein produced by the genes for sensitivity to 5-FU, whereas the RT-PCR determination measures the level of mRNA. This does not seem to be a drawback since it has been shown that in colorectal and gastric tumors the level of mRNA is closely correlated with the level of protein produced (4, 5).

The calibration range used in our study was prepared from a line of colo-rectal cancer cells (WiDr). Although they are not of the same histological type as epidermoid carcinomas of the pharyngolarynx, we used them as the reference. The values obtained were in fact expressed in an arbitrary relative unit (ARU), so that only a comparison of the different values was meaningful, the long-term aim being to establish an expression value that would distinguish between responders and non responders.

Our main assessment criterion was based on the clinical assessment after C3 by a panendoscopy and a cervical CT scan. This was the same as the criterion used by Etienne et al. (12, 13). The panendoscopy was not ideal since it was partly subjective. In order to increase its reliability, the lesion was tattooed during the initial panendoscopy and the 2 panendoscopies (initial and after C3) were carried out by the same operator.

We think that other factors may also be involved in the response to chemotherapy, including other genes for sensitivity to 5-FU, the associated platinum salt, interindividual variability of the pharmacokinetics of 5-FU and the patient’s general condition. Orotate phosphoribosyl transferase (OPRT) is an enzyme corresponding to the alternative pathway to that of TS for the anabolism of 5-FU. The study of Ichikawa et al. carried out on metastatic colo-rectal cancers, concludes that the level of expression of mRNA of OPRT could be an index of the efficacy of the chemotherapy (14). All our patients were treated with a combination of 5-FU/platinum salt. The platinum salt probably contributes to the response to chemotherapy, even though it is accepted that 5-FU plays the predominant role in this combination (15, 16). Excision Repair Cross Complementing 1 (ERCC1), is an enzyme involved in the excision-repair of the anomalies created by the insertion of platinum compounds into the DNA helix. According to the study of Metzger et al. (17), carried out on gastric cancers, the expression of ERCC1 and of TS in the tumor are two independent variables, correlated with the clinical response to chemotherapy combining 5-FU and cisplatin. Furthermore, dosage adaptation of 5-FU seems to be interesting due to a high degree of interindividual variability in the clearance of the drug (18). An essential factor in the variability of the pharmacokinetics of 5-FU is the activity of DPD. This activity has been shown to display a circadian rhythm, which is responsible for an increase in the plasma concentrations of 5-FU during the night, during continuous infusions (19). Finally the patient’s general status probably plays a non-negligible role in the response to chemotherapy, however, we think that its role in our study was limited, since all the patients included displayed a general condition compatible with beginning chemotherapy (WHO Performance Status ≤ 2 or Karnofsky index > 60%).

## Conclusion

This study is intended to contribute to establishing the pharmacogenetics, which in turn is intended to establish the individual profile of the response to chemotherapy. The preliminary findings of this prospective study are interesting and encourage us to continue by extending it to other sensitivity genes, in particular OPRT and ERCC1, and through the use of the multiple microdisplay of gene expression.

## Figures and Tables

**Figure 4:**
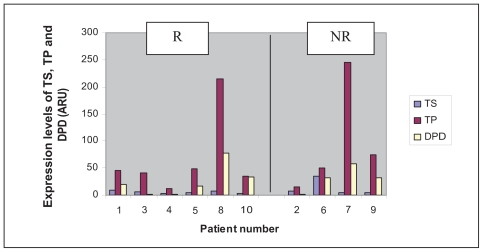
Expression levels of TS, TP and DPD (ARU) depending on clinical response after C3: patient responder (R) or non-responder (NR).

**Table 1:** Specific primers used for PCR (bp: base pair).

Gene	Primers	Fragment amplified (bp)
G6PDH	Sense: 5′-TggAgAATgAgAggTggg ATg-3′ Antisense: 5′-gAgCTTCACgTTCTTgTATCTgT-3′	269 bp
TS	Sense: 5′-AgCCTgAgAgATgAATTCCC-3′ Antisense: 5′-CTCTggTggAgAATCCCAgg-3′	184 bp
TP	Sense: 5′-CAgATCggggCCATgCTgAT-3′ Antisense: 5′-ATCCAAggTgCCTCCTgTgTg-3′	261 bp
DPD	Sense: 5′-gATTCTggCTACCAggCTATACA-3′ Antisense: 5′-CCggATTCACAgATAAgggTACg-3′	178 bp

**Table 2:** WHO criteria for the response to chemotherapy.

Response	Definition
Complete response (CR)	Complete disappearance of the tumor visible or palpable
Partial response (RP)	Reduction in the size of the tumor by at least 50%
Stable disease (SD)	No response or progression of the tumor
Progressive disease (PD)	Increase by more than 25% in the size of the tumor

**Table 3:** Description of the population.

Identification number	Sex (a)	Age (years)	Site (b)	Histology (c)	TNM	No. courses	Outcome after C3 (d)	Treatment after C3 (e)
1	M	49	Larynx G	1	T3 N2a M0	3	PR	TPL + RX
2	M	75	Epiglottis	2	T3 N3 M0	4	PD	C4 + RX
3	M	44	R Larynx	1	T3 N0 M0	3	PR	TPL + RX
4	M	65	R Piriform Sinus	2	T3 N0 M0	3	RC	RX
5	M	51	L Piriform Sinus	1	T3 N2a M0	3	PR	TPL + RX
6	M	76	L Larynx	2	T4 N0 M0	3	SD	RX
7	M	60	R Piriform Sinus	2	T4 N3 M0	1	PD	TPL + RX
8	M	49	R Larynx	1	T3 N0 M0	3	PR	TPL + RX
9	M	75	R Piriform Sinus	2	T3 N2b M0	6	PD	C6 + RX
10	M	41	L Piriform Sinus	2	T3 N3 M0	3	PR	TPL + RX

(a): sex; M: male. (b): tumor site; R: right; L: left. (c): Histology; 1: clearly-differentiated epidermoid carcinoma; 2: moderately or slightly differentiated epidermoid carcinoma. (d): outcome after C3; PR: partial response; CR: complete response; PD: progressive disease; SD: stable disease. (e): Treatment after C3; TPL: total pharyngo-laryngectomy; RX: radiotherapy; Cx: xth course of chemotherapy.

**Table 4:** Distribution of the patients on the basis of their TNM classification and clinical response to neo-adjuvant chemotherapy.

	PR	CR	SD	PD	Total
T3 N0 M0	2	1	0	0	3
T3 N2a M0	2	0	0	0	2
T3 N2b M0	1	0	0	1	2
T3 N3 M0	0	0	0	1	1
T4 N0 M0	0	0	1	0	1
T4 N3 M0	0	0	0	1	1
Total	5	1	1	3	10
